# Impact of integrated medication management program on medication errors in a medical center: an interrupted time series study

**DOI:** 10.1186/s12913-022-08178-w

**Published:** 2022-06-20

**Authors:** Kuan-Lin Chen, Chih-Fen Hunag, Wang-Huei Sheng, Yu-Kuei Chen, Chi-Chuan Wang, Li-Jiuan Shen

**Affiliations:** 1 Graduate Institute of Clinical Pharmacy, College of Medicine, National Taiwan University, Taipei, Taiwan; 2 Department of Pharmacy, National Taiwan University Hospital, Taipei, Taiwan; 3 School of Pharmacy, College of Medicine, National Taiwan University, Taipei, Taiwan; 4 Department of Internal Medicine, National Taiwan University Hospital, Taipei, Taiwan; 5 Department of Nursing, National Taiwan University Hospital, Taipei, Taiwan

**Keywords:** Clinical pharmacy, Interrupted time series, Pharmaceutical services

## Abstract

**Background:**

Medication errors (MEs) are harmful to patients during hospitalization, especially elderly patients. To reduce MEs, an integrated medication management (IMM) model was developed in a 2500-bed medical center, allowing a clinical pharmacist to participate in the daily ward round and perform medication reconciliation and medication reviews. This study aimed to evaluate the impact of the IMM model on MEs and medication utilization using a quasi-experimental design.

**Methods:**

We conducted an interrupted time-series study using the aggregated data of monthly admissions from two wards of a medical center, where one ward served as the intervention and the other served as the external control. The pre- and post-intervention phases comprised of 40 and 12 monthly observational units, respectively. The primary outcome was the mean number of ME reports, which were further investigated for different ME types. The mean number of daily inpatient prescriptions, mean number of daily self-prepared medications, and median daily medication costs were measured. All outcomes were measured per admission episode. Segmented regression was used to evaluate the level and slope changes in the outcomes after IMM model implementation, and subgroup analyses were performed to examine the effects on different groups.

**Results:**

After IMM model implementation, the mean number of ME reports increased (level change: 1.02, 95% confidence interval [CI]: 0.68 to 1.35, *P* < 0.001). The number of reports has shown a dramatic increase in omissions or medication discrepancies, inappropriate drug choices, and inappropriate routes or formulations. Furthermore, the mean number of daily inpatient prescriptions was reduced for patients aged ≥75 years (level change: −1.78, 95% CI: −3.06 to −0.50, *P* = 0.009). No significant level or slope change was observed in the control ward during the post-intervention phase.

**Conclusions:**

The IMM model improved patient safety and optimized medication utilization by increasing the reporting of MEs and decreasing the number of medications used.

**Supplementary Information:**

The online version contains supplementary material available at 10.1186/s12913-022-08178-w.

## Background

A medication error (ME) is defined as “a failure in the treatment process that leads to, or has the potential to lead to, harm to the patient” and is usually preventable [1]. During hospitalization, approximately 6% of patients have experienced MEs; some of these MEs have been observed to turn into adverse drug events and result in a prolonged length of stay (LOS) or even death [2]. The elderly are especially at a high risk for ME because they often have multiple morbidities and are already on multiple medications [3, 4]. Moreover, a systematic review published in 2017 revealed that MEs might increase the economic burden, with the cost of an ME during hospitalization ranging from EUR 17.6 to EUR 6432.16 [5]. As MEs are common in practice and potentially harmful to patients, the reduction of MEs has become a challenge for the healthcare system.

MEs can occur during any part of the drug therapy process, including prescription, transcription, dispensation, or administration [1]. Numerous clinical pharmaceutical services (CPS) have been developed to prevent these errors. According to previous studies, CPS not only reduces MEs, but also decreases the medication costs, prevents adverse drug events, and improves other quality indicators [6–8]. However, many of these studies have been conducted under highly controlled conditions. Additionally, the efficacy of CPS may be influenced by several factors, such as labor and workflow. Therefore, the results of previous studies cannot sufficiently reflect the effectiveness of CPS in real-world settings.

To reduce MEs and improve the quality of care, we propose an integrated CPS model called the National Taiwan University Hospital Integrated Medication Management (NTUH-IMM) model. This study aimed to evaluate the effectiveness of the NTUH-IMM model on MEs and medication utilization in routine clinical practice using a quasi-experimental design.

## Methods

### Study design and settings

To evaluate the effectiveness of the NTUH-IMM model in a real-world setting, we applied an interrupted time series (ITS) design. The ITS design is a quasi-experimental method used to examine the differences in the outcomes between different periods (time series) using longitudinal data [9].

This study was conducted in the Division of Multidisciplinary Medicine (DMM) of the NTUH, a 2500-bed medical center in Taiwan. There were two wards in the DMM with similar settings, making them suitable for conducting a quasi-experimental study. We implemented the NTUH-IMM model in one of the DMM wards (i.e., the intervention ward) in July 2018 and considered the other as the control ward. As an ITS is usually conducted on a single group, it could avoid issues due to unequal distribution between groups, such as between-group selection bias or unmeasured confounders. However, history, defined as an independent concurrent event with the intervention that potentially influences the outcomes, has been considered as the major threat to any ITS design. Therefore, we introduced an external control group as a counterfactual to address this issue [10].

The two wards, with 36 and 35 beds, respectively, were on the same floor. Five attending physicians, 12 nurse practitioners, 18 nurses, and one central pharmacy pharmacist were allocated to each ward. The staff were rotated between the two study wards during the pre-intervention period, but not the post-intervention period. As usual care was implemented in both study wards before the intervention; therefore, staff rotation should not have affected the pre-intervention estimates.

The patients admitted to the DMM were mainly transferred from the emergency department and presented with acute illnesses. Over 60% of these patients were at least 65 years old, with poor functional status (Barthel’s score, mean and standard deviation: 61 ± 35) and high comorbidity (Charlson score, mean and standard deviation: 3.7 ± 3.4) [11]. The transfer to the DMM was decided by managers who were blind to and independent of this study. No randomization was performed.

Since elderly individuals were more vulnerable to MEs, we chose the DMM wards, with the majority of patients being at least 65 years of age, to implement the intervention and conduct the study. Due to the fact that we also intended to promote the NTUH-IMM model to other wards at the NTUH, the elements of the NTUH-IMM model were not specifically designed for the elderly.

The study period included a 40-month pre-intervention phase (phase 1: January 1, 2015 to April 30, 2018) and a 12-month post-intervention phase (phase 2: July 1, 2018 to June 30, 2019). The crossover period was from May 1, 2018 to June 30, 2018. The patients could have been exposed to the usual care and the new intervention during this period; thus, we did not consider admissions during the crossover period.

### Data sources

We used the NTUH Integrated Medical Database (NTUH-iMD) and NTUH Pharmaceutical Service Record Database (NTUH-PSRD) as the data sources. Both databases contained data generated at the NTUH and only de-identified information was available to the researchers. The NTUH-iMD included records from outpatient and inpatient visits at the NTUH, including demographics, diagnosis, laboratory data, and medication records.

The NTUH-PSRD provides records of ME reports from the internal reporting system at the NTUH. All the pharmacists at NTUH voluntarily reported MEs to the reporting system when they detected MEs and made suggestions. The number of ME reports is one of the references used for performance evaluation. Moreover, ME reports should be verified by an independent clinical pharmacist after they have been documented. The content of each ME report included the types of ME, rationale for being regarded as an error, recommendation to correct the error, and whether the recommendation was accepted or not by the prescriber.

Although MEs can occur in any phase of medication use, such as prescription, dispensation, and administration, only the MEs related to prescriptions can be reported to the NTUH reporting system with fixed types. The ME types and their definitions are presented in **Table A**1. For example, when a pharmacist found that a medication was provided at an incorrect concentration that was different from the prescription, the pharmacist was not able to report this error because it was not a prescription error.

### Interventions

The NTUH-IMM model was developed to reduce the MEs occurring in patients and optimize medication utilization. It was based on the integrated medication management services developed by Scullin et al. [12], with some modifications. The ‘integrated’ part of the NTUH-IMM model was extended in two aspects: (1) integration of a clinical pharmacist, who was not included in the DMM wards, to participate in the activities of the medical team and (2) coherent integration of the process of medication reconciliation and medication review.

### Implementation preparation

Before the implementation of the NTUH-IMM model in the intervention ward, the clinical pharmacist had to receive a 6-month interdisciplinary-collaboration training, which contained an internship with other experienced clinical pharmacists in different wards. In addition, an explanation session aimed at informing other healthcare professionals about the change and potential benefits held in the intervention ward to facilitate the implementation of the NTUH-IMM model. The standard processes of the NTUH-IMM model were co-developed with the clinical pharmacist and central pharmacy pharmacist, who would execute these interventions.

The details of the NTUH-IMM model are described below, and a comparison between the NTUH-IMM model and usual care is presented in Table 1**.**

**Table 1:** Differences between the NTUH-IMM model and usual care

	The NTUH-IMM model	Usual care
**Pharmacist integrated into the medical team**		
Clinical pharmacists participated in the daily ward round	Yes	No
Clinical pharmacists stay on the ward to provide services	Yes	No
Communication between pharmacists and other healthcare professionals	Mainly face-to-face	Mainly by telephone or text messages
**Medication reconciliation during admission**		
Medication history documentation	•The central pharmacy pharmacist interviews the patients or caregivers to collect medication history •Medication history was further verified with data from the PharmaCloud system •Best possible medication history was documented on the EMR^a^ system with the details of using-pattern	•The nurse practitioner interviews the patients or caregivers to collect the medication history •Medication history was not verified •Medication history was documented on the EMR system without details
Reconciliation	•The clinical pharmacist and the central pharmacy pharmacist discuss the patients’ medication history •If medication discrepancy exists, the clinical pharmacist reconciles it according to the patient’s condition	•The pharmacists are not required to perform medication reconciliation routinely •There is no standard process for pharmacists to perform medication reconciliation
**Medication review during hospitalization**		
Data resource	•The best possible medication history •Direct observe the patients •EMR system •Information from other healthcare professionals	•EMR system
Decision making	•The clinical pharmacist discusses the regimens with other healthcare professionals and make a decision collaboratively	•The central pharmacy pharmacist makes suggestions to healthcare professionals without comprehensive discussion

^a^ *EMR* Electronic medical records

#### Medication reconciliation during admission

When a patient was admitted to the intervention ward, a central pharmacy pharmacist interviewed the patient or the caregiver within 3 days to collect the medication history and information on allergies, difficulty in taking medication, adherence, and supplement/herb/non-prescription drug use. The medication history was further verified with data from the PharmaCloud system, a cloud-based inquiry system maintained by the National Health Insurance (NHI) Administration in Taiwan since 2013, which provided the prescription drug records of patients over the past 3 months [13]. All the information mentioned above was documented as the best possible medication history (BPMH) in the electronic medical record (EMR) system at the NTUH and could be accessed by all healthcare providers of the patient.

Moreover, the central pharmacy pharmacist compared the admission medication list with the BPMH to identify whether a medication discrepancy existed. If so, the central pharmacy pharmacist would inform and discuss it with the clinical pharmacist, who would further reconcile it according to the patient’s condition.

#### Medication review during hospitalization

During the hospital stay of the patient, the clinical pharmacist performed a medication review to optimize the medication regimen of the patient periodically. Several aspects were considered, such as the patient demographics, medication history, treatment response, therapeutic goal, laboratory values, and patient preference. Additionally, the clinical pharmacist surveyed drug-drug interactions, drug-food interactions, contraindications, and the potential causes of adverse events in the medication regimen of the patient.

To gather information and provide timely recommendations, the clinical pharmacist participated in daily ward rounds with the medical team, observed the patient directly, and exchanged opinions with other healthcare professionals. When the patient’s regimen required modification, the clinical pharmacist could discuss it with the medical team and make decisions collaboratively. After the ward round, the clinical pharmacist stayed at the nursing station to provide medication consultation services to other healthcare professionals.

### Study sample

Admissions to the two DMM wards during the study period were included if they met the following criteria: (1) the age of the patient was a minimum of 20 years at admission, (2) direct transfer from the emergency department, (3) LOS of at least 2 days, and (4) at least 180 days apart for two consecutive admissions to the study wards. There were no specific exclusion criteria. All the patients admitted during the study period participated in this study; thus, no specific sampling technique was implemented.

### Measures

We retrieved the demographic variables and primary diagnoses of the patients at admission from the NTUH-iMD; additionally, these data were used to represent the baseline characteristics. For the ITS design, we aggregated the data from patients admitted to the study wards in each calendar month into an observational unit. For instance, if a patient was admitted on May 30, 2018 and discharged on June 10, 2018, the data were placed into an observational unit representing May 2018. The outcome variables were measured for each admission episode.

The primary outcome was the mean number of ME reports as the NTUH-IMM model was developed to reduce the MEs in patients. Since a higher number of MEs reported by pharmacists meant that more MEs were detected and corrected, we assumed that the elevation of the mean number of ME reports indicated a reduction in MEs occurring to the patients. To ensure the validity of the reports, only when the recommendation in the ME reports was accepted by the prescribers would the reports be adopted for this study. We further investigate the effects of the NTUH-IMM model on different types of ME reports. The definitions of each ME type are listed in **Table A****1**. The mean numbers of each type of ME report during phases 1 and 2 were measured, and the post-pre ratio was calculated by dividing the number of reports in phase 2 by those in phase 1.

To further understand the effectiveness of NTUH-IMM on medication use during hospitalization, the following outcome variables were also included: the mean number of daily inpatient prescriptions (IPs), mean number of daily self-prepared medications (SPMs), and median daily medication cost. The medication cost was converted from NTD to USD, according to the exchange rate on June 28, 2019 (NTD: USD = 1:0.033). These outcome variables were selected and evaluated because it was assumed that the NTUH-IMM model would decrease the number of unnecessary medications and could further reduce the medication costs of the patients during hospitalization.

The definitions of the outcome variables are presented in **Table A****2**.

### Statistical analysis

Descriptive statistics and chi-square tests were used to describe the baseline characteristics. We used a segmented regression analysis to examine the level or slope changes in the outcome variables between phases 1 and 2. The linear regression model was expressed as follows: Y_t_ = b_0_ + b_1_*T_t_ + b_2_*X_t_ + b_3_*XT_t_. Here, Y_t_ is the outcome value at time t and T_t_ is a continuous variable that indicates the time series at time t. Notably, X_t_ is a dummy variable indicating the implementation of the NTUH-IMM model at time t, and XT_t_ is a continuous variable that denotes the time series after the NTUH-IMM implementation at time t (XT_t_ = 0 before the NTUH-IMM implementation). The estimation of b_1_ refers to the trend of the outcome value without any intervention effect, the estimation of b_2_ refers to the intervention effect on the level of outcome value immediately after the intervention implementation, and the estimation of b_3_ refers to the intervention effect on the trend of outcome value after the intervention implementation.

We used an autoregressive error model to adjust the autocorrelation of the regression model. Segmented regression analyses were applied to both the intervention and control wards. The outcome changes in the intervention ward were interpreted as the effects of the NTUH-IMM model, whereas those in the control ward were interpreted as counterfactuals. All the analyses were conducted using SAS version 9.4, and the significance threshold was set to a *P*-value< 0.05.

We also conducted a sensitivity analysis by shortening the data collection interval to half a month, as the power of the ITS depends on the number of observational units. Furthermore, we conducted several subgroup analyses to evaluate the potential effect modification by including patients with different characteristics. First, we restricted the admissions to late-elderly patients (aged 75+ years) to determine whether the effects of the NTUH-IMM model were modified by age. Second, we focused on admissions without prolonged LOS (≥ 30 days). Extant studies have revealed that the LOS highly depends on the destination of post-discharge care, and delayed discharge might be due to a lack of patient resources to facilitate discharge [14, 15]. Therefore, medication for admission with prolonged LOS might be stabilized after the acute problem is solved, and the effects of the NTUH-IMM model might be modified by prolonged LOS. Third, we removed the admissions of patients who died or were transferred to the intensive care unit (ICU), as these patients likely had poor prognoses and would not benefit from the intervention.

## Results

A total of 5610 admissions were included in the analysis, with 2816 admissions in the intervention ward and 2794 in the control ward. The baseline characteristics are presented in **Tables A****3** and **A****4****.** Only the top five most frequent primary diagnoses, which constituted 30–40% of all primary diagnoses, were analysed, while all other primary diagnoses accounted for less than 3%. First, we compared the baseline characteristics between phases 1 and 2 in the intervention and control wards. The intervention ward showed a lower proportion of patients who were women, had a primary diagnosis of fever, and had a higher proportion of primary diagnosis of pneumonia in phase 2, whereas there were no significant differences in the baseline characteristics in the control ward (**Table A****3**). Subsequently, we compared the intervention and control wards in different phases and found no significant differences in the baseline characteristics except for sex (**Table A****4**).

**Fig. 1:**
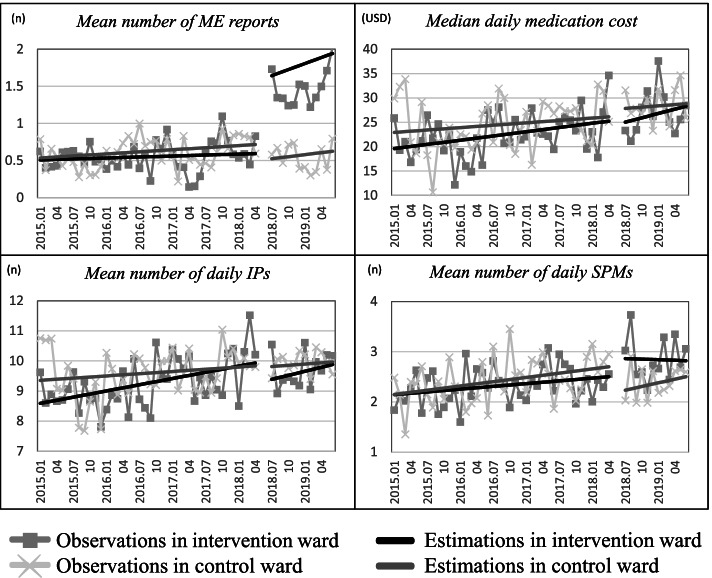
Regression estimation and observed time series of the outcome variables. ME, medication error; IPs, inpatient prescriptions; SPMs, self-prepared medications; USD, United States dollar

The time series and estimation of the outcome variables from the intervention and control wards are shown in Fig. 1**.** The estimated changes in the level and slope for each outcome variable are listed in Table 2. As the results from the control ward served as counterfactuals and the outcome variables did not change significantly with respect to the level or slope, we focused on the results for the intervention ward.

**Table 2:** Effects of the NTUH-IMM model on monthly-measured outcome variables

Outcome variables	Predictor variables	Intervention Ward				Control Ward			
		Coefficient of determination	Estimation (SE)	95% CI	***P***-value	Coefficient of determination	Estimation (SE)	95% CI	***P***-value
**Mean number of ME reports**	**Intercept**	0.90	0.51 (0.07)	(0.36 to 0.65)	< 0.001	0.40	0.51 (0.04)	(0.44 to 0.58)	< 0.001
	**Time**		0.00 (0.00)	(0.00 to 0.01)	0.480		0.00 (0.00)	(0.00 to 0.01)	0.021
	**Intervention**		1.02 (0.16)	(0.70 to 1.34)	< 0.001		−0.15 (0.12)	(−0.38 to 0.08)	0.208
	**Time after intervention**		0.03 (0.02)	(−0.02 to 0.07)	0.231		0.00 (0.01)	(−0.03 to 0.03)	0.871
**Mean number of daily IPs**	**Intercept**	0.26	8.56 (0.23)	(8.12 to 9.00)	< 0.001	0.17	9.34 (0.32)	(8.71 to 9.97)	< 0.001
	**Time**		0.03 (0.01)	(0.02 to 0.05)	0.001		0.01 (0.01)	(−0.01 to 0.04)	0.369
	**Intervention**		−0.59 (0.49)	(−1.55 to 0.37)	0.235		−0.03 (0.62)	(−1.26 to 1.19)	0.957
	**Time after intervention**		0.01 (0.06)	(−0.11 to 0.13)	0.862		0.00 (0.08)	(−0.15 to 0.15)	0.984
**Mean number of daily SPMs**	**Intercept**	0.32	2.13 (0.12)	(1.90 to 2.37)	< 0.001	0.14	2.13 (0.13)	(1.89 to 2.38)	< 0.001
	**Time**		0.01 (0.01)	(0.00 to 0.02)	0.076		0.01 (0.01)	(0.00 to 0.02)	0.011
	**Intervention**		0.37 (0.26)	(−0.14 to 0.87)	0.164		−0.49 (0.27)	(−1.03 to 0.04)	0.078
	**Time after intervention**		−0.01 (0.03)	(−0.07 to 0.05)	0.680		0.01 (0.03)	(−0.05 to 0.08)	0.758
**Median daily medication cost**	**Intercept**	0.27	19.45 (1.36)	(16.78 to 22.12)	< 0.001	0.14	22.85 (1.58)	(19.76 to 25.93)	< 0.001
	**Time**		0.14 (0.06)	(0.03 to 0.26)	0.016		0.08 (0.07)	(− 0.05 to 0.21)	0.228
	**Intervention**		−0.48 (2.96)	(−6.29 to 5.33)	0.871		1.65 (3.42)	(−5.06 to 8.36)	0.632
	**Time after intervention**		0.15 (0.36)	(−0.55 to 0.85)	0.679		0.00 (0.41)	(−0.81 to 0.82)	0.991

*SE* Standard error, *CI* Confidence interval, *ME* Medication error, *IPs* Inpatient prescriptions, *SPMs* Self-prepared medications

Segment linear regression was used to model the correlation between the longitudinal outcome variable and independent variables (time, intervention, and time after intervention), and the model was as follows: Y_t_ = b_0_ + b_1_*T_t_ + b_2_*X_t_ + b_3_*XT_t_. The estimations of time, intervention, and time after intervention indicate the point estimations of b_1_, b_2_, and b_3,_ respectively

### Medication errors

The mean number of ME reports was approximately 0.51 and remained constant in phase 1, but increased immediately after the intervention by 1.02 (95% confidence interval [CI]: 0.70 to 1.34, *P* < 0.001). However, the slope of the mean number of ME reports showed no significant change during the study period (Table 2).

**Fig. 2:**
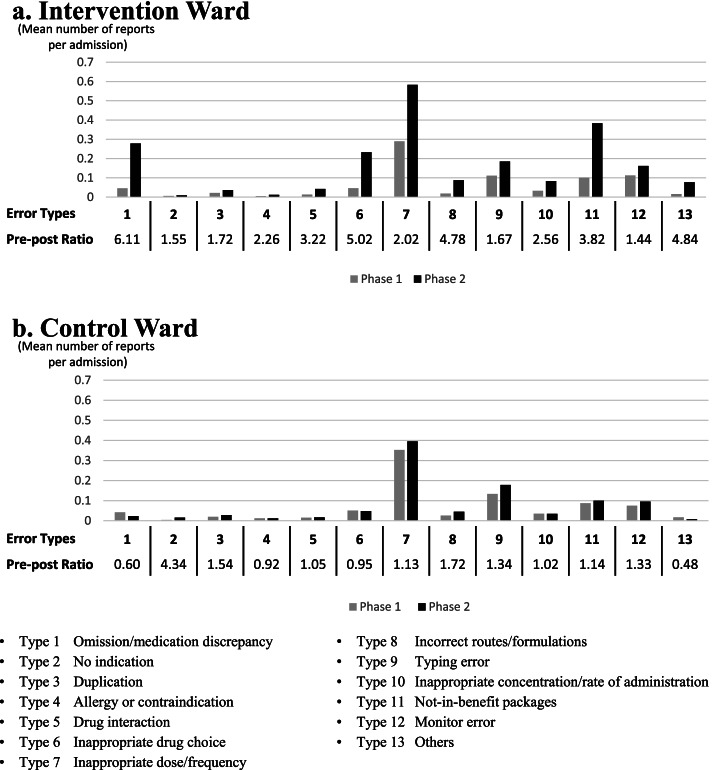
Mean number of medication error reports by type in phases 1 and 2

When we investigated ME reports of different types between phases 1 and 2, the mean number of ME reports of the following types presented significantly elevated levels: omissions or medication discrepancies, inappropriate doses or frequencies, typing errors, inappropriate concentrations or rates of administration, and not-in-benefit packages. Moreover, the mean number of ME reports of the following types showed a slope escalation: inappropriate drug choices, inappropriate routes or formulations, and others. However, no significant level or slope changes were observed for no indication, duplication, allergy or contraindication, drug interaction, or monitor error (Table 3). Notably, the post-pre ratios were 6.11 for omissions or medication discrepancies, 5.02 for inappropriate drug choices, 4.78 for inappropriate route or formulations, and 3.82 for the not-in-benefit package (Fig. 2).

**Table 3:** Effects of the NTUH-IMM model on the different types of medication error reports

Outcome variables	Predictor variables	Intervention Ward				Control Ward			
		Coefficient of determination	Estimation (SE)	95% CI	***P***-value	Coefficient of determination	Estimation (SE)	95% CI	***P***-value
**Omission/medication discrepancy**	**Intercept**	0.85	0.036 (0.014)	(0.008 to 0.064)	< 0.001	0.08	0.038 (0.010)	(0.019 to 0.058)	< 0.001
	**Time**		0.000 (0.001)	(−0.001 to 0.002)	0.429		0.000 (0.000)	(−0.001 to 0.001)	0.828
	**Intervention**		0.194 (0.031)	(0.134 to 0.254)	< 0.001		−0.036 (0.022)	(−0.079 to 0.007)	0.105
	**Time after intervention**		0.005 (0.004)	(−0.002 to 0.012)	0.193		0.002 (0.003)	(−0.003 to 0.008)	0.360
**No indication**	**Intercept**	0.31	0.008 (0.002)	(0.004 to 0.011)	0.000	0.39	0.003 (0.002)	(−0.001 to 0.007)	0.186
	**Time**		0.000 (0.000)	(0.000 to 0.000)	0.199		0.000 (0.000)	(0.000 to 0.000)	0.931
	**Intervention**		0.004 (0.005)	(−0.006 to 0.014)	0.475		0.025 (0.005)	(0.015 to 0.035)	< 0.001
	**Time after intervention**		0.001 (0.001)	(−0.001 to 0.002)	0.333		−0.002 (0.001)	(−0.003 to − 0.001)	0.001
**Duplication**	**Intercept**	0.18	0.017 (0.007)	(0.003 to 0.030)	0.018	0.10	0.022 (0.006)	(0.011 to 0.033)	< 0.001
	**Time**		0.000 (0.000)	(0.000 to 0.001)	0.589		0.000 (0.000)	(−0.001 to 0.000)	0.221
	**Intervention**		−0.005 (0.015)	(− 0.034 to 0.024)	0.739		0.015 (0.012)	(−0.009 to 0.039)	0.232
	**Time after intervention**		0.003 (0.002)	(−0.001 to 0.006)	0.111		0.001 (0.001)	(−0.002 to 0.004)	0.659
**Allergy or contraindication**	**Intercept**	0.28	0.001 (0.002)	(−0.003 to 0.005)	0.624	0.08	0.005 (0.005)	(−0.004 to 0.014)	0.279
	**Time**		0.000 (0.000)	(0.000 to 0.000)	0.025		0.000 (0.000)	(0.000 to 0.001)	0.185
	**Intervention**		0.000 (0.006)	(−0.011 to 0.011)	1.000		−0.018 (0.01)	(−0.038 to 0.002)	0.082
	**Time after intervention**		0.000 (0.001)	(−0.001 to 0.001)	0.920		0.002 (0.001)	(−0.001 to 0.004)	0.217
**Drug- interaction**	**Intercept**	0.33	0.007 (0.007)	(− 0.006 to 0.020)	0.304	0.02	0.016 (0.005)	(0.006 to 0.027)	0.004
	**Time**		0.000 (0.000)	(0.000 to 0.001)	0.346		0.000 (0.000)	(0.000 to 0.000)	0.977
	**Intervention**		0.015 (0.015)	(−0.014 to 0.043)	0.308		0.008 (0.012)	(−0.014 to 0.031)	0.475
	**Time after intervention**		0.001 (0.002)	(−0.002 to 0.005)	0.432		−0.001 (0.001)	(−0.004 to 0.001)	0.310
**Inappropriate choice of drug**	**Intercept**	0.77	0.052 (0.016)	(0.020 to 0.083)	0.002	0.00	0.046 (0.015)	(0.016 to 0.075)	0.004
	**Time**		0.000 (0.001)	(−0.002 to 0.001)	0.716		0.000 (0.001)	(−0.001 to 0.001)	0.761
	**Intervention**		0.074 (0.035)	(0.006 to 0.143)	0.039		−0.01 (0.033)	(−0.074 to 0.055)	0.773
	**Time after intervention**		0.020 (0.004)	(0.011 to 0.028)	< 0.001		0.000 (0.004)	(−0.008 to 0.008)	0.994
**Inappropriate dose/frequency**	**Intercept**	0.65	0.327 (0.030)	(0.268 to 0.386)	< 0.001	0.04	0.324 (0.036)	(0.254 to 0.394)	< 0.001
	**Time**		−0.002 (0.001)	(− 0.004 to 0.001)	0.214		0.001 (0.002)	(−0.002 to 0.004)	0.579
	**Intervention**		0.301 (0.065)	(0.173 to 0.429)	< 0.001		0.061 (0.077)	(−0.091 to 0.213)	0.433
	**Time after intervention**		0.005 (0.008)	(−0.011 to 0.02)	0.544		−0.007 (0.009)	(−0.025 to 0.011)	0.453
**Inappropriate route/formulation**	**Intercept**	0.75	0.015 (0.004)	(0.007 to 0.023)	0.001	0.30	0.022 (0.006)	(0.010 to 0.033)	< 0.001
	**Time**		0.000 (0.000)	(0.000 to 0.001)	0.287		0.000 (0.000)	(0.000 to 0.001)	0.487
	**Intervention**		0.003 (0.011)	(−0.019 to 0.024)	0.810		0.043 (0.016)	(0.012 to 0.074)	0.010
	**Time after intervention**		0.010 (0.001)	(0.007 to 0.013)	< 0.001		−0.005 (0.002)	(−0.009 to − 0.001)	0.017
**Typing error**	**Intercept**	0.60	0.139 (0.006)	(0.126 to 0.151)	< 0.001	0.11	0.108 (0.022)	(0.066 to 0.151)	< 0.001
	**Time**		−0.001 (0.000)	(−0.002 to − 0.001)	< 0.001		0.001 (0.001)	(− 0.001 to 0.003)	0.240
	**Intervention**		0.122 (0.022)	(0.079 to 0.166)	< 0.001		−0.012 (0.047)	(−0.104 to 0.080)	0.797
	**Time after intervention**		−0.004 (0.003)	(−0.009 to 0.002)	0.233		0.004 (0.006)	(−0.007 to 0.015)	0.509
**Inappropriate concentration/ rate of administration**	**Intercept**	0.41	0.048 (0.010)	(0.028 to 0.067)	< 0.001	0.13	0.012 (0.010)	(−0.007 to 0.032)	0.224
	**Time**		−0.001 (0.000)	(−0.001 to 0.000)	0.121		0.001 (0.000)	(0.000 to 0.002)	0.012
	**Intervention**		0.112 (0.021)	(0.070 to 0.154)	< 0.001		−0.013 (0.022)	(−0.055 to 0.030)	0.567
	**Time after intervention**		−0.007 (0.003)	(−0.012 to − 0.002)	0.008		− 0.003 (0.003)	(− 0.008 to 0.002)	0.247
**Not-in-benefit package**	**Intercept**	0.84	0.073 (0.014)	(0.046 to 0.101)	< 0.001	0.22	0.074 (0.015)	(0.044 to 0.104)	< 0.001
	**Time**		0.001 (0.001)	(0.000 to 0.002)	0.037		0.001 (0.001)	(−0.001 to 0.002)	0.375
	**Intervention**		0.236 (0.038)	(0.161 to 0.311)	< 0.001		−0.028 (0.038)	(−0.103 to 0.047)	0.472
	**Time after intervention**		0.001 (0.005)	(−0.009 to 0.010)	0.902		0.002 (0.005)	(−0.007 to 0.011)	0.651
**Monitor error**	**Intercept**	0.23	0.071 (0.022)	(0.028 to 0.113)	0.002	0.06	0.059 (0.017)	(0.025 to 0.093)	0.001
	**Time**		0.002 (0.001)	(0.000 to 0.004)	0.028		0.001 (0.001)	(−0.001 to 0.002)	0.325
	**Intervention**		0.018 (0.057)	(−0.094 to 0.130)	0.752		0.031 (0.037)	(−0.041 to 0.104)	0.403
	**Time after intervention**		−0.006 (0.007)	(−0.020 to 0.008)	0.410		−0.005 (0.004)	(−0.014 to 0.004)	0.290
**Others**	**Intercept**	0.68	0.008 (0.008)	(−0.008 to 0.025)	0.340	0.04	0.014 (0.006)	(0.001 to 0.026)	0.038
	**Time**		0.000 (0.000)	(0.000 to 0.001)	0.239		0.000 (0.000)	(0.000 to 0.001)	0.587
	**Intervention**		0.013 (0.013)	(−0.012 to 0.038)	0.315		−0.015 (0.014)	(−0.042 to 0.013)	0.306
	**Time after intervention**		0.006 (0.002)	(0.002 to 0.009)	0.001		0.000 (0.002)	(−0.003 to 0.004)	0.891

*SE* Standard error, *CI* Confidence interval

The outcome variables in Table 3 are the number of ME reports of different types, as shown in Table A1

The operational definition of these outcome variables is as follows:(∑ Number of each type of ME report in an admission)/(number of admissions per observational unit). A segment linear regression was used to model the correlation between the longitudinal outcome variable and independent variables (time, intervention, and time after intervention), and the model was as follows: Yt = b0 + b1*Tt + b2*Xt + b3*XT_t._ The estimations of time, intervention, and time after intervention indicate the point estimations of b1, b2, and b3, respectively

### Number of medications

The mean number of daily IPs and SPMs were 8.56 and 2.13 at baseline, respectively. The slope of the mean number of daily IPs increased slowly every month, with a slope of 0.03 (95% CI: 0.02 to 0.05, *P* = 0.001), while the mean number of daily SPMs did not change during the study period. However, implementation of the NTUH-IMM model did not significantly affect the level and slope of the mean number of daily IPs and SPMs (Table 2).

### Medication cost

The baseline of the median daily medication cost was USD 19.45 and increased continuously at the rate of USD 0.14 per month (95% CI: 0.03 to 0.26, *P* = 0.016) during the study period. There was no level or slope change in the median daily medication cost after the implementation of the NTUH-IMM model (Table 2).

### Sensitivity analyses

When we shortened the observational interval to half a month, the observational units increased from 40 to 80 in Phase 1 and from 12 to 24 in Phase 2. However, the direction and size of the effects of the NTUH-IMM model on each outcome variable remained similar to those of the main analysis (**Table A****5**).

### Subgroup analyses

When we restricted admission to late-elderly patients, different results were obtained **(**Table 4). The level of the mean number of daily IPs dropped significantly by 1.78 (95% CI: − 3.06 to − 0.50, *P* = 0.009) after the NTUH-IMM model was implemented, while no significant change was found in the main analysis. The level of median daily medication cost also decreased by USD 5.90; however, this change was not statistically significant (95% CI: − 12.31 to 0.51, *P* = 0.078). When we restricted admissions to those without a prolonged LOS or those who did not expire or were transferred to the ICUs during hospitalization, the results were similar to those of the main analysis (**Tables A****6** and **A****7**).

**Table 4:** Subgroup analysis: Effects of the NTUH-IMM model on outcome variables in late-elderly patients

Outcome variables	Predictor variables	Intervention Ward				Control Ward			
		Coefficient of determination	Estimation (SE)	95% CI	***P***-value	Coefficient of determination	Estimation (SE)	95% CI	***P***-value
**Mean number of ME reports**	**Intercept**	0.74	0.45 (0.07)	(0.32 to 0.59)	< 0.001	0.35	0.53 (0.07)	(0.40 to 0.66)	< 0.001
	**Time**		0.01 (0.00)	(0.00 to 0.01)	0.068		0.01 (0.00)	(0.00 to 0.01)	0.007
	**Intervention**		0.71 (0.18)	(0.35 to 1.07)	< 0.001		−0.22 (0.19)	(− 0.59 to 0.15)	0.246
	**Time after intervention**		0.02 (0.02)	(−0.03 to 0.06)	0.442		−0.02 (0.02)	(−0.07 to 0.03)	0.454
**Mean number of daily IPs**	**Intercept**	0.27	8.44 (0.30)	(7.85 to 9.03)	< 0.001	0.04	9.37 (0.31)	(8.77 to 9.98)	< 0.001
	**Time**		0.05 (0.01)	(0.03 to 0.08)	< 0.001		0.02 (0.01)	(−0.01 to 0.04)	0.261
	**Intervention**		−1.78 (0.65)	(−3.06 to −0.50)	0.009		−0.44 (0.67)	(−1.75 to 0.88)	0.521
	**Time after intervention**		0.04 (0.08)	(−0.11 to 0.20)	0.594		0.03 (0.08)	(−0.13 to 0.19)	0.717
**Mean number of daily SPMs**	**Intercept**	0.33	2.59 (0.13)	(2.33 to 2.84)	< 0.001	0.06	2.66 (0.18)	(2.30 to 3.01)	< 0.001
	**Time**		0.00 (0.01)	(−0.01 to 0.01)	0.712		0.01 (0.01))	(0.00 to 0.03)	0.136
	**Intervention**		0.56 (0.35)	(−0.13 to 1.25)	0.119		−0.49 (0.39)	(−1.26 to 0.28)	0.217
	**Time after intervention**		−0.01 (0.04)	(−0.10 to 0.07)	0.756		0.03 (0.05)	(−0.06 to 0.12)	0.542
**Median daily medication cost**	**Intercept**	0.24	16.58 (1.5)	(13.63 to 19.53)	< 0.001	0.08	21.43 (2.16)	(17.2 to 25.65)	< 0.001
	**Time**		0.18 (0.06)	(0.06 to 0.31)	0.007		0.02 (0.09)	(−0.16 to 0.20)	0.838
	**Intervention**		−5.90 (3.27)	(−12.31 to 0.51)	0.078		3.36 (4.69)	(−5.82 to 12.55)	0.476
	**Time after intervention**		0.58 (0.40)	(−0.19 to 1.36)	0.148		0.09 (0.57)	(−1.02 to 1.20)	0.876

*SE* Standard error, *CI* Confidence interval, *ME* Medication error, *IPs* Inpatient prescriptions, *SPMs* Self-prepared medications

In this subgroup analysis, we restricted the admissions to patients aged ≥75 years. Segment linear regression was used to model the correlation between the longitudinal outcome variable and independent variables (time, intervention, and time after intervention), and the model was as follows: Y_t_ = b_0_ + b_1_*T_t_ + b_2_*X_t_ + b_3_*XT_t_. The estimations of time, intervention, and time after intervention indicate the point estimations of b_1_, b_2_, and b_3,_ respectively

## Discussion

The results support that NTUH-IMM increases the detection and reporting of MEs significantly in hospitalized patients, consistent with prior studies investigating the effects of pharmacist intervention on MEs [16, 17]. Moreover, the number of medications used by late-elderly patients decreased after the implementation of the NTUH-IMM model.

Reliable information is the cornerstone of high-quality CPS [18–20]. Therefore, in the NTUH-IMM model, we included several strategies to enable clinical pharmacists to collect more comprehensive medical information than in the case of usual care. First, the NTUH-IMM model requires the clinical pharmacist to participate in medical teams and ward activities. Therefore, the clinical pharmacist can gather medical information from the EMRs and communicate directly with other healthcare professionals. Considering that medical information is not routinely documented and records may be incomplete or inaccurate in EMRs systems [21, 22], direct interaction with other healthcare professionals is another way to retrieve those pieces of information.

Second, unlike usual care, in the NTUH-IMM model, a central pharmacy pharmacist conducted patient interviews to complete the BPMH, and provided these records to the clinical pharmacist. The NTUH-IMM model allows the clinical pharmacist to retrieve additional details on the types of medications and their administration before admission. Several studies have also supported that pharmacist-led medication reconciliation provides a more accurate patient medication history and identifies more medication discrepancies [23–25].

Finally, the NTUH-IMM model integrates two pharmacists to perform different parts of CPS. For example, the central pharmacist interviewed the patients and the clinical pharmacist reconciled the medication discrepancies when performing medication reconciliation. These assignments enabled pharmacists to accomplish care tasks and collect medical information more efficiently.

The number of ME reports were observed to increase after we introduced the NTUH-IMM model, whereas the post-pre ratios differed according to the type of ME reports. According to our results, the reported MEs classified as omissions or medication discrepancies increased by more than six times (from a mean of 0.045 to 0.278 per admission). This is consistent with a previous study showing that the pharmacists’ collaboration with other healthcare professionals increases the reporting of medication omission errors [26]. Our results imply that the clinical pharmacist in the NTUH-IMM model received more information about the current status and previous medication history of the patients on admission than under usual care. Considering that approximately 30% of MEs occur during the transition of care, medication reconciliation on admission is crucial and can affect the subsequent hospital course [27]. In general, medication discrepancies can be defined as intended or unintended, and unintended discrepancies are usually regarded as MEs [28]. It is difficult for a pharmacist to distinguish between intended and unintended medication discrepancies solely by referring to EMRs. However, the clinical pharmacist in the NTUH-IMM model could assess whether a medication discrepancy was intended via direct communication with prescribers.

Notably, two types of reported MEs, inappropriate drug choices and not-in-benefit packages, also increased dramatically from a mean of 0.046 to 0.233 per admission and from a mean of 0.100 to 0.383 per admission, respectively. These ME types indicate that the patients have an indication, but the prescribed medication is inappropriate or not covered by the NHI benefits in Taiwan. A pharmacist cannot decide whether the medication in use is appropriate without sufficient medical information because any medication can be used for several indications and the clinical signs and symptoms of the patients are diverse. Under the NTUH-IMM model, the clinical pharmacist participated in ward rounds to directly observe and interact with patients and to better evaluate the appropriateness of medication use.

From the subgroup analysis, the mean daily IPs decreased in late-elderly patients after implementing the NTUH-IMM model. This is probably because the complex regimen for late-elderly patients with multiple morbidities has more room for improvement. Polypharmacy is a critical problem in elderly patients and is associated with multimorbidity [29, 30], and the incidence of unnecessary medication use is higher in patients with polypharmacy [31]. Based on previous research, up to 44% of hospitalized elderly patients have at least one unnecessary medication at discharge [32]. In our study, the prevalence of excessive polypharmacy (defined as using more than 10 medications) in late-elderly patients was 68.4% in Phase 1 and 74.8% in Phase 2 (data not shown here). Therefore, we expect that the late-elderly have a higher risk of unnecessary medication use, meaning that our intervention can reduce the number of medications.

As the NTUH-IMM model decreased the number of daily IPs for late-elderly patients, we assumed that it could further reduce the daily medication costs. Although the reduction in daily medication costs was not statistically significant, the estimated change for late-elderly patients was larger than that in the main analysis. Furthermore, reducing inappropriate drug choices and doses or frequencies could potentially reduce the medication costs. For example, when a late-elderly patient is treated with antibiotics, the clinical pharmacist might recommend switching to an agent with a narrower spectrum, lowering the dose, or even ceasing it according to the patient’s clinical situation, which could result in a decrease in the cost of medication. Although central pharmacy pharmacists could also recommend prescribers to adjust regimens, they are not able to provide timely responses; additionally, the lack of a collaborative relationship between the central pharmacy pharmacists and other healthcare professionals might impede the application of the recommendation.

To the best of our knowledge, few studies have investigated the impact of a service model on ME reporting and medication utilization in a real-world setting. The ITS design used in this study may further inform researchers who want to examine their services using longitudinal data. As the outcome variables were all potentially confounded by time-varying variables, such as autocorrelation or maturation, we conducted an ITS analysis with segment regression and autoregressive error models to address these problems. Furthermore, we introduced an external control to avoid history threats. The allocation of patients was assumed to be similar to randomization, as the managers were blinded to the study. Sensitivity and subgroup analyses were also conducted to confirm the results and address any effect modifications.

This study has a few limitations. First, only prescription errors were recorded in NTUH-PSRD. However, the NTUH-IMM model can logically reduce administration and transcription errors by integrating a clinical pharmacist into the medical team. Therefore, the actual effect of the NTUH-IMM model on ME reporting should be larger if administration and transcription errors were considered. Second, the study was not blinded to the pharmacists in the intervention ward; therefore, they might have made extra efforts to detect and report MEs. Even though the study did not provide additional rewards to pharmacists, an overestimated effect of the NTUH-IMM model on the number of ME reports is anticipated. Third, our study might be underpowered for some outcomes, given that the effect size of the NTUH-IMM model might be small for the mean number of IPs and SPMs and median medication cost. The power of ITS depends on the number of observational units. We had limited observational units with a relatively short observation period in phase 2, and we might not have been able to observe the long-term effect of the NTUH-IMM. Nevertheless, the number of observational units in our study met the general requirement (at least 12 points before and after the intervention) [33]. Finally, although the medication cost was based on the NHI reimbursement price in November 2020, the reimbursement price may change over time.

To further support the findings of this study, future studies could evaluate the impacts of IMM model implementation in a larger population or a different setting. Future pharmacoeconomics studies may also be conducted to confirm the effects of CPS on medical costs.

## Conclusions

These results support our intervention, which helps lower MEs in hospitalized patients and reduces the number of medications used for late-elderly patients. Therefore, we recommend that hospitals include clinical pharmacists in their medical teams to perform CPS, which can improve patient safety and optimize medication utilization.

## Supplementary Information

**Additional file 1: Table A1.** Definitions of the different types of medication errors in the reporting system at the NTUH. **Table A2.** Outcome variable definitions. **Table A3.** Baseline characteristics of the patients between phases 1 and 2. **Table A4.** Baseline characteristics of the patients between the intervention and control wards. **Table A5.** Sensitivity analysis: effects of the NTUH-IMM model on outcome variables when shortening the observational interval to half-month. **Table A6.** Subgroup analysis: Effects of the NTUH-IMM model on the outcome variables in patients without prolonged lengths of stay. **Table A7.** Subgroup analysis: Effects of the NTUH-IMM model on the outcome variables for patients who did not expire or were transferred to the intensive care unit during hospitalization

## Data Availability

The datasets generated and analyzed during the current study are not publicly available due to following reasons. First, the data are originated from electronic medical records at NTUH, and they contain sensitive patient information at both the patient and the institution levels. According to the “Personal Information Protection Act”, the availability of medical records is restricted by the government of Taiwan. Thus, the data cannot be distributed publicly. Second, according to the policy and regulations of the data use agreement, the data are only available to the employees of NTUH; using data for other purposes require a direct approval from the Department of Medical Research, NTUH. The authors have no right to share the data without the permission from the Department of Medical Research, NTUH. To request access of the data, please contact Department of Medical Research, NTUH directly at cmrd@ntuh.gov.tw.

## Abbreviation

CPS: Clinical pharmaceutical service
DMM: Division of Multidisciplinary Medicine
EMR: Electronic medical record
ICU: Intensive care unit
IP: Inpatient prescription
ITS: Interrupted time series
LOS: Length of stay
ME: Medication error
NHI: National Health Insurance
NTUH: National Taiwan University Hospital
NTUH-iMD: NTUH Integrated Medical Database
NTUH-IMM: NTUH Integrated Medication Management
NTUH-PSRD: NTUH Pharmaceutical Service Record Database
NTD: New Taiwan dollar
SPM: Self-prepared medication
USD: United States dollar
