# Supplementary material for: Impact of integrated medication management program on medication errors in a medical center: an interrupted time series study
Source: BMC Health Serv Res. 2022 Jun 20;22:796. doi: 10.1186/s12913-022-08178-w (PMC9210585; doi:10.1186/s12913-022-08178-w)
Supplement: Supplementary file 1 — Additional file 1: Table A1. Definitions of the different types of medication errors in the reporting system at the NTUH. Table A2. Outcome variable definitions. Table A3. Baseline characteristics of the patients between phases 1 and 2. Table A4. Baseline characteristics of the patients between the intervention and control wards. Table A5. Sensitivity analysis: effects of the NTUH-IMM model on outcome variables when shortening the observational interval to half-month. Table A6. Subgroup analysis: Effects of the NTUH-IMM model on the outcome variables in patients without prolonged lengths of stay. Table A7. Subgroup analysis: Effects of the NTUH-IMM model on the outcome variables for patients who did not expire or were transferred to the intensive care unit during hospitalization [file 12913_2022_8178_MOESM1_ESM.docx]

**Appendix**

**Table A1. Definitions of the different types of medication errors in the reporting system at the NTUH**

| **Types of medication errors** | **Definitions** |
| --- | --- |
| **Omission/medication discrepancy** | - Omitting treatment for patients with indications - Un-intended medication discrepancies |
| **No indication** | Use of a prescription drug without an indication |
| **Duplication** | Unnecessarily combining two drugs to treat an indication |
| **Allergy or contraindication** | Use of a prescription drug on patients with known allergy or contraindication |
| **Drug interactions** | Prescription of a drug that interacts with the current drugs |
| **Inappropriate drug choice** | Better alternatives or substitutions for the current therapy |
| **Inappropriate dose/frequency** | Drugs with inappropriate dose or frequency |
| **Inappropriate route/formulation** | Drugs with inappropriate route or formulation |
| **Typing error** | Prescription with typing error |
| **Inappropriate concentration/rate of administration** | Drugs with inappropriate concentration or rate of administration |
| **Not-in-benefit packages** | Prescribing a drug not covered by the NHI benefit package |
| **Monitor error** | Not monitoring patients with high risk medication appropriately |
| **Others** | Medication errors not described above |

NHI: National Health Insurance

**Table A2. Outcome variable definitions**

| **Outcome variables** | **Operational definition** | **Note** |
| --- | --- | --- |
| Mean number of ME reports | $\frac{\sum Number of ME reports in an admission}{Number of admissions per observational unit}$ | - |
| Mean number of daily IPs | $\frac{\sum\frac{Days supply of each IP}{LOS} in an admission}{Number of admissions per observational unit}$ | Our hospital allows patients to bring their own medications; however, the medications should be managed by healthcare professionals during hospitalization |
| Mean number of daily SPMs | $\frac{\sum\frac{Days supply of each SPM}{LOS} in an admission}{Number of admissions per observational unit}$ |  |
| Median daily medication cost | Median number of  $\frac{\sum Quantiity of medication used*price}{LOS}$ in the admission  to each observational unit | The price was based on the NHI reimbursement price in November 2020.  The SPMs were not calculated into the daily medication cost, as they were paid before the hospital course |

ME, medication error; IPs, inpatient prescriptions; SPMs, self-prepared medications; NHI, National Health Insurance

**Table A3. Baseline characteristics of the patients between phases 1 and 2**

|  | **Intervention Ward** | | | **Control Ward** | | |
| --- | --- | --- | --- | --- | --- | --- |
|  | Phase 1  (n=2130) | Phase 2  (n=686) | *P*-value | Phase 1  (n=2155) | Phase 2  (n=639) | *P*-value |
| **Age>=75** (%) | 984 (46.20) | 327 (47.67) | 0.134 | 1000 (46.40) | 298 (46.64) | 0.189 |
| **65<=Age<75** (%) | 443 (20.80) | 159 (23.18) |  | 428 (19.86) | 145 (22.69) |  |
| **Age<65** (%) | 703 (33.00) | 200 (29.15) |  | 727 (33.74) | 196 (30.67) |  |
| **Female** (%) | 1070 (50.23) | 301 (43.88) | 0.004 | 967 (44.87) | 299 (46.79) | 0.392 |
| **Primary diagnosis** |  |  |  |  |  |  |
| **Pneumonia** (%) | 335 (15.73) | 130 (18.95) | 0.048 | 335 (15.55) | 104 (16.28) | 0.656 |
| **Fever** (%) | 165 (7.75) | 37 (5.39) | 0.038 | 186 (8.63) | 40 (6.26) | 0.054 |
| **UTI** (%) | 134 (6.29) | 40 (5.83) | 0.663 | 132 (6.13) | 38 (5.95) | 0.868 |
| **GI bleeding** (%) | 105 (4.93) | 38 (5.54) | 0.527 | 129 (5.99) | 33 (5.16) | 0.435 |
| **HF exacerbation** (%) | 52 (2.44) | 12 (1.75) | 0.290 | 73 (3.39) | 13 (2.03) | 0.082 |

UTI: urinary tract infection; HF: heart failure, GI: gastrointestinal

**Table A4. Baseline characteristics of the patients between the intervention and control wards**

|  | **Phase 1** | | | **Phase 2** | | |
| --- | --- | --- | --- | --- | --- | --- |
|  | Intervention  ward  (n=2130) | Control  ward  (n=2155) | *P*-value | Intervention  ward  (n=686) | Control  ward  (n=639) | *P*-value |
| **Age>=75** (%) | 984 (46.20) | 1,000 (46.40) | 0.725 | 327 (47.67) | 298 (46.64) | 0.834 |
| **65<=Age<75** (%) | 443 (20.80) | 428 (19.86) |  | 159 (23.18) | 145 (22.69) |  |
| **Age<65** (%) | 703 (33.00) | 727 (33.74) |  | 200 (29.15) | 196 (30.67) |  |
| **Female** (%) | 1070 (50.23) | 967 (44.87) | <0.001 | 301 (43.88) | 299 (46.79) | 0.287 |
| **Primary diagnosis** |  |  |  |  |  |  |
| **Pneumonia** (%) | 335 (15.73) | 335 (15.55) | 0.869 | 130 (18.95) | 104 (16.28) | 0.202 |
| **Fever** (%) | 165 (7.75) | 186 (8.63) | 0.291 | 37 (5.39) | 40 (6.26) | 0.501 |
| **UTI** (%) | 134 (6.29) | 132 (6.13) | 0.822 | 40 (5.83) | 38 (5.95) | 0.929 |
| **GI bleeding** (%) | 105 (4.93) | 129 (5.99) | 0.128 | 38 (5.54) | 33 (5.16) | 0.762 |
| **HF exacerbation** (%) | 52 (2.44) | 73 (3.39) | 0.066 | 12 (1.75) | 13 (2.03) | 0.703 |

UTI: urinary tract infection; HF: heart failure, GI: gastrointestinal

**Table A5. Sensitivity analysis: effects of the NTUH-IMM model on outcome variables when shortening the observational interval to half-month**

| **Outcome variables** | **Predictor variables** | **Intervention Ward** | | | | **Control Ward** | | | |
| --- | --- | --- | --- | --- | --- | --- | --- | --- | --- |
|  |  | **Coefficient of determination** | **Estimation (SE)** | **95% CI** | ***P*-value** | **Coefficient of determination** | **Estimation (SE)** | **95% CI** | ***P*-value** |
| **Mean number of ME reports** | **Intercept** | 0.83 | 0.50 (0.05) | (0.40 to 0.59) | <0.001 | 0.12 | 0.52 (0.05) | (0.43 to 0.61) | <0.001 |
|  | **Time** |  | 0.00 (0.00) | (0.00 to 0.00) | 0.281 |  | 0.00 (0.00) | (0.00 to 0.00) | 0.072 |
|  | **Intervention** |  | 1.14 (0.15) | (0.86 to 1.43) | <0.001 |  | -0.13 (0.12) | (-0.36 to 0.09) | 0.250 |
|  | **Time after intervention** |  | 0.00 (0.01) | (-0.01 to 0.02) | 0.607 |  | 0.00 (0.01) | (-0.02 to 0.01) | 0.832 |
| **Mean number of daily IPs** | **Intercept** | 0.16 | 8.50 (0.22) | (8.07 to 8.94) | <0.001 | 0.18 | 9.28 (0.22) | (8.86 to 9.71) | <0.001 |
|  | **Time** |  | 0.02 (0.00) | (0.01 to 0.03) | <0.001 |  | 0.01 (0.00) | (0.00 to 0.02) | 0.159 |
|  | **Intervention** |  | -0.58 (0.48) | (-1.51 to 0.35) | 0.227 |  | 0.28 (0.55) | (-0.79 to 1.35) | 0.610 |
|  | **Time after intervention** |  | 0.01 (0.03) | (-0.05 to 0.06) | 0.847 |  | -0.02 (0.04) | (-0.09 to 0.05) | 0.576 |
| **Mean number of daily SPMs** | **Intercept** | 0.18 | 2.17 (0.13) | (1.91 to 2.43) | <0.001 | 0.07 | 2.16 (0.13) | (1.91 to 2.42) | <0.001 |
|  | **Time** |  | 0.00 (0.00) | (0.00 to 0.01) | 0.167 |  | 0.01 (0.00) | (0.00 to 0.01) | 0.011 |
|  | **Intervention** |  | 0.29 (0.28) | (-0.26 to 0.84) | 0.308 |  | -0.52 (0.28) | (-1.06 to 0.03) | 0.065 |
|  | **Time after intervention** |  | 0.01 (0.02) | (-0.03 to 0.04) | 0.644 |  | 0.00 (0.02) | (-0.03 to 0.04) | 0.788 |
| **Median daily medication cost** | **Intercept** | 0.21 | 19.99 (1.3) | (17.45 to 22.53) | <0.001 | 0.16 | 21.24 (1.22) | (18.85 to 23.62) | <0.001 |
|  | **Time** |  | 0.06 (0.03) | (0.01 to 0.11) | 0.034 |  | 0.09 (0.03) | (0.03 to 0.14) | 0.002 |
|  | **Intervention** |  | -0.52 (2.84) | (-6.08 to 5.04) | 0.855 |  | -0.52 (3.1) | (-6.6 to 5.56) | 0.868 |
|  | **Time after intervention** |  | 0.14 (0.18) | (-0.21 to 0.49) | 0.430 |  | -0.02 (0.2) | (-0.41 to 0.36) | 0.907 |

SE, standard error; CI, confidence interval; ME, medication error; IPs, inpatient prescriptions; SPMs, self-prepared medications

In this sensitivity analysis, we shortened the data collection interval to half a month, to increase the number of observational units. Segment linear regression was used to model the correlation between the longitudinal outcome variable and independent variables (time, intervention, and time after intervention), and the model was as follows: Yt=b0+b1*Tt+b2*Xt+b3*XT_t_. The estimations of time, intervention, and time after intervention indicate the point estimations of b1, b2, and b3, respectively.

**Table A6. Subgroup analysis: Effects of the NTUH-IMM model on the outcome variables in patients without prolonged lengths of stay**

| **Outcome variables** | **Predictor variables** | **Intervention Ward** | | | | **Control Ward** | | | |
| --- | --- | --- | --- | --- | --- | --- | --- | --- | --- |
|  |  | **Coefficient of determination** | **Estimation (SE)** | **95% CI** | ***P*-value** | **Coefficient of determination** | **Estimation (SE)** | **95% CI** | ***P*-value** |
| **Mean number of ME reports** | **Intercept** | 0.89 | 0.33 (0.05) | (0.24 to 0.42) | <0.001 | 0.03 | 0.44 (0.05) | (0.35 to 0.54) | <0.001 |
|  | **Time** |  | 0.00 (0.00) | (0.00 to 0.01) | 0.057 |  | 0.00 (0.00) | (0.00 to 0.00) | 0.732 |
|  | **Intervention** |  | 0.78 (0.12) | (0.55 to 1.01) | <0.001 |  | -0.12 (0.11) | (-0.33 to 0.09) | 0.260 |
|  | **Time after intervention** |  | 0.00 (0.01) | (-0.02 to 0.03) | 0.812 |  | 0.01 (0.01) | (-0.01 to 0.04) | 0.330 |
| **Mean number of daily IPs** | **Intercept** | 0.27 | 7.94 (0.21) | (7.53 to 8.35) | <0.001 | 0.06 | 8.95 (0.22) | (8.51 to 9.39) | <0.001 |
|  | **Time** |  | 0.04 (0.01) | (0.02 to 0.05) | <0.001 |  | 0.00 (0.01) | (-0.01 to 0.02) | 0.643 |
|  | **Intervention** |  | -0.72 (0.46) | (-1.61 to 0.18) | 0.123 |  | 0.17 (0.49) | (-0.79 to 1.12) | 0.731 |
|  | **Time after intervention** |  | 0.00 (0.06) | (-0.11 to 0.11) | 0.992 |  | 0.02 (0.06) | (-0.10 to 0.13) | 0.794 |
| **Mean number of daily SPMs** | **Intercept** | 0.34 | 2.24 (0.13) | (1.98 to 2.50) | <0.001 | 0.16 | 2.23 (0.14) | (1.96 to 2.50) | <0.001 |
|  | **Time** |  | 0.01 (0.01) | (0.00 to 0.02) | 0.048 |  | 0.02 (0.01) | (0.01 to 0.03) | 0.007 |
|  | **Intervention** |  | 0.26 (0.29) | (-0.30 to 0.82) | 0.367 |  | -0.60 (0.30) | (-1.18 to -0.01) | 0.051 |
|  | **Time after intervention** |  | 0.01 (0.03) | (-0.06 to 0.07) | 0.884 |  | 0.01 (0.03) | (-0.06 to 0.08) | 0.758 |
| **Median daily medication cost** | **Intercept** | 0.26 | 17.82 (1.29) | (15.29 to 20.34) | <0.001 | 0.35 | 20.58 (0.94) | (18.73 to 22.43) | <0.001 |
|  | **Time** |  | 0.11 (0.05) | (0.01 to 0.22) | 0.041 |  | 0.09 (0.04) | (0.01 to 0.17) | 0.041 |
|  | **Intervention** |  | 0.56 (2.8) | (-4.93 to 6.05) | 0.842 |  | 3.05 (3.07) | (-2.98 to 9.07) | 0.327 |
|  | **Time after intervention** |  | 0.11 (0.34) | (-0.55 to 0.78) | 0.744 |  | -0.25 (0.39) | (-1.01 to 0.52) | 0.532 |

SE, standard error; CI, confidence interval; ME, medication error; IPs, inpatient prescriptions; SPMs, self-prepared medications

In this subgroup analysis, we focused on admissions whose length of hospital stay was < 30 days. A segment linear regression was used to model the correlation between the longitudinal outcome variable and independent variables (time, intervention, and time after intervention), and the model was as follows: Yt=b0+b1*Tt+b2*Xt+b3*XT_t_. The estimations of time, intervention, and time after intervention indicate the point estimations of b1, b2, and b3, respectively.

**Table A7. Subgroup analysis: Effects of the NTUH-IMM model on the outcome variables for patients who did not expire or were transferred to the intensive care unit during hospitalization**

| **Outcome variables** | **Predictor variables** | **Intervention Ward** | | | | **Control Ward** | | | |
| --- | --- | --- | --- | --- | --- | --- | --- | --- | --- |
|  |  | **Coefficient of determination** | **Estimation (SE)** | **95% CI** | ***P*-value** | **Coefficient of determination** | **Estimation (SE)** | **95% CI** | ***P*-value** |
| **Mean number of ME reports** | **Intercept** | 0.87 | 0.44 (0.06) | (0.33 to 0.55) | <0.001 | 0.27 | 0.47 (0.04) | (0.39 to 0.54) | <0.001 |
|  | **Time** |  | 0.00 (0.00) | (0.00 to 0.01) | 0.221 |  | 0.00 (0.00) | (0.00 to 0.01) | 0.060 |
|  | **Intervention** |  | 0.97 (0.14) | (0.69 to 1.24) | <0.001 |  | -0.17 (0.12) | (-0.39 to 0.06) | 0.160 |
|  | **Time after intervention** |  | 0.03 (0.02) | (0.00 to 0.07) | 0.078 |  | 0.01 (0.01) | (-0.02 to 0.04) | 0.507 |
| **Mean number of daily IPs** | **Intercept** | 0.19 | 8.20 (0.23) | (7.76 to 8.64) | <0.001 | 0.06 | 8.80 (0.25) | (8.30 to 9.30) | <0.001 |
|  | **Time** |  | 0.03 (0.01) | (0.01 to 0.05) | 0.005 |  | 0.01 (0.01) | (-0.01 to 0.03) | 0.244 |
|  | **Intervention** |  | -0.48 (0.49) | (-1.44 to 0.48) | 0.334 |  | -0.06 (0.55) | (-1.14 to 1.02) | 0.917 |
|  | **Time after intervention** |  | 0.00 (0.06) | (-0.11 to 0.12) | 0.945 |  | 0.00 (0.07) | (-0.13 to 0.13) | 0.975 |
| **Mean number of daily SPMs** | **Intercept** | 0.32 | 2.27 (0.13) | (2.02 to 2.52) | <0.001 | 0.12 | 2.31 (0.14) | (2.04 to 2.58) | <0.001 |
|  | **Time** |  | 0.01 (0.01) | (0.00 to 0.02) | 0.067 |  | 0.01 (0.01) | (0.00 to 0.03) | 0.021 |
|  | **Intervention** |  | 0.50 (0.27) | (-0.03 to 1.04) | 0.072 |  | -0.54 (0.30) | (-1.13 to 0.05) | 0.077 |
|  | **Time after intervention** |  | -0.04 (0.03) | (-0.10 to 0.03) | 0.289 |  | 0.02 (0.04) | (-0.05 to 0.09) | 0.625 |
| **Median daily medication cost** | **Intercept** | 0.16 | 18.39 (1.4) | (15.65 to 21.14) | <0.001 | 0.06 | 21.49 (1.64) | (18.28 to 24.7) | <0.001 |
|  | **Time** |  | 0.08 (0.06) | (-0.03 to 0.20) | 0.164 |  | 0.06 (0.07) | (-0.07 to 0.20) | 0.363 |
|  | **Intervention** |  | 0.67 (3.05) | (-5.3 to 6.64) | 0.827 |  | 0.45 (3.56) | (-6.53 to 7.43) | 0.900 |
|  | **Time after intervention** |  | 0.10 (0.37) | (-0.62 to 0.83) | 0.778 |  | 0.04 (0.43) | (-0.8 to 0.88) | 0.926 |

SE, standard error; CI, confidence interval; ME, medication error; IPs, inpatient prescriptions; SPMs, self-prepared medications

In this subgroup analysis, we excluded the admissions of patients who died or were transferred to intensive care units. A segment linear regression was used to model the correlation between the longitudinal outcome variable and independent variables (time, intervention, and time after intervention), and the model was as follows: Yt=b0+b1*Tt+b2*Xt+b3*XT_t_. The estimations of time, intervention, and time after intervention indicate the point estimations of b1, b2, and b3, respectively
